# Research hotspots and trends in the interaction mechanisms of neuroinflammation and sleep disorders: A bibliometric analysis based on WOS

**DOI:** 10.1016/j.ibneur.2025.08.011

**Published:** 2025-08-15

**Authors:** Nan Zhao, Zhao-qiong Zhu, Qihai Gong, Rui Jiang

**Affiliations:** a Department of Anesthesiology, Affiliated Stomatological Hospital of Zunyi Medical University, Zunyi 563003, China; b Early Clinical Research Ward, Affliated Hospital of Zunyi Medical University, Zunyi 563003, China; c Department of Anesthesiology, Affiliated Hospital of Zunyi Medical University, Zunyi 563003, China; d Key Laboratory of Basic Pharmacology of Ministry of Education and Joint International Research Laboratory of Ethnomedicine of Ministry of Education, Zunyi Medical University, Zunyi 563006, China

**Keywords:** Neuroinflammation, Sleep disorders, Bibliometrics, CiteSpace, VOSviewer

## Abstract

This study aims to analyze the research hotspots and trends regarding neuroinflammation in sleep disorders over the past 30 years through bibliometric and review analyses. Relevant publications were sourced from the Web of Science Core Collection (WoSCC). We utilized VOSviewer and CiteSpace for the visualization and quantitative analysis of the literature to provide an objective presentation and predictions. A total of 2545 publications related to neuroinflammation and sleep disorders were identified, with the overall number of publications showing a continuous upward trend. Most of the publications originated from the United States and China. The University of Toronto, Harvard Medical School, and the University of California, Los Angeles, are leading institutions in this field. David Gozal and Michael R. Irwin are recognized as prominent figures in this area. The *International Journal on Molecular Sciences* and *Brain Behavior and Immunity* are the journals with the highest publication volume. Keywords and clustering analyses indicate that the current research in this field has developed a multidisciplinary integration pattern, with core trends focusing on the multi-axis regulation of neuroimmune interaction mechanisms, as well as individualized targeted intervention strategies based on biomarkers and gene editing. Additionally, the development of emerging technologies such as organoids and the establishment of multidisciplinary collaborative networks bring new hope for exploring the interactions between neuroinflammation and sleep disorders.

## Introduction

Sleep is a universal and essential physiological process for living organisms, crucial for energy restoration, immune regulation, cognitive enhancement, and emotional adjustment (Siegel, 2022). Sleep disorders refer to a range of abnormal sleep states, including difficulty falling asleep, decreased sleep quality, reduced sleep duration, abnormal behaviors during sleep, and disruptions in the normal alternation of sleep-wake rhythms (Lane et al., 2023). With the accelerated pace of society and increased life pressures, the issue of sleep disorders has become increasingly prominent. As a common chronic condition, the prevalence of sleep disorders in European countries is approximately 10 % of the total population (Riemann et al., 2017). Epidemiological surveys in the United States indicate that 27.3 % of adults reported insomnia in the 12 months prior to the survey, with the resulting health economic burden significantly higher than that of 18 other conditions, including arthritis, depression, and hypertension (Olfson et al., 2018). According to the "White Paper on Sleep Health of Chinese Residents" published by the China Sleep Research Society in 2024, 64 % of respondents reported sleep disorders, and 22 % of the population reported poor sleep quality. The most common symptoms include difficulty falling asleep and waking up too early. Sleep disorders not only disrupt metabolic processes, endocrine functions, and the nervous system, but also increase the risk of related diseases such as cardiovascular diseases, diabetes, neurodegenerative disorders, depression, anxiety, and cancer, severely affecting individuals' physical and mental health as well as their quality of life. Therefore, improving sleep quality has become a primary research focus for scientists worldwide (Shen et al., 2023, Abelleira et al., 2024, Lacerda et al., 2024).

Fortunately, an increasing number of scholars are recognizing the importance of sleep and conducting research on this topic. In recent years, researchers have comprehensively explored the prevalence, influencing factors, and adverse consequences of sleep disorders. Interventions aimed at addressing sleep disorders have shown some effectiveness, yet the mechanisms underlying the health issues caused by sleep disorders remain controversial (Ahn et al., 2024, Chen et al., 2025, Chennaoui et al., 2020). Increasing evidence suggests a close relationship between neuroinflammation and the pathophysiology of sleep disorders. Research findings indicate that sleep disorders can promote the expression of complement components C3 and C5 in the hippocampus, activating immune cells in the central nervous system (CNS), particularly the activation of microglia and astrocytes, leading to the release of pro-inflammatory factors and exacerbation of neuroinflammation (Gudkov et al., 2023, Zhai et al., 2023). Meanwhile, the activation of immune cells and the release of pro-inflammatory factors can also promote cell apoptosis and the phagocytosis of neurons. Furthermore, neuroinflammation can inversely regulate sleep by affecting endogenous sleep-regulating molecules such as adenosine and cytokines (Sang et al., 2023, Thondala et al., 2024). In short, there is a bidirectional relationship between sleep disorders and neuroinflammation. As research into neuroinflammation and sleep disorders deepens, this field is gaining increasing attention from researchers. Although the research field has entered a phase of "mechanism-technology-clinical" spiral development, there are still bottlenecks that need to be addressed, such as unclear mechanisms of organ interaction networks, insufficient sensitivity and specificity of biomarkers, immature targeted precision treatment technologies, and the difficulty of existing models in simulating the chronic processes of human sleep-inflammation interactions. Therefore, scientifically summarizing the literature in this field and elucidating the role of neuroinflammation in the occurrence and progression of sleep disorders is of great significance for future medical research.

Bibliometric analysis involves searching for and collecting relevant literature in a specific research field, using mathematical and statistical methods to conduct qualitative and quantitative evaluations of the research literature from multiple perspectives, including different countries/regions, authors, institutions, journals, keywords, and references. Notably, it can visually present the current status, hotspots, and development trends of research, as well as predict future research directions (Hassan and Duarte, 2024). Therefore, in order to comprehensively reference the current research trends in sleep disorders and neuroinflammation, this study used literature sourced from the Web of Science Core Collection (WoSCC) and employed bibliometric tools to organize and visualize the related research over the past thirty years. The aim is to provide insights and references for subsequent research and clinical applications in this field.

## Materials and methods

### Data source and retrieval strategy

Literatures related to sleep disorders and neuroinflammation were extracted from the WoSCC database. Being one of the largest and most widely used database in the world, WoSCC provides the latest and reliable information with authority and reference significance. The retrieval strategy of this study was: TS= (“sleep disorder*” OR “sleep disturbance*” OR “insomnia” OR “sleep apnea” OR “sleep deprivation” OR “sleep insufficiency” OR “difficulty falling asleep” OR “trouble staying asleep” OR “interrupted sleep”) AND TS= (“neuroinflammation*” OR “neurogenic inflammati*” OR “nervous inflammat*” OR “central inflammat*” OR “brain inflammat*”). The language was limited to "English", and the literature types were limited to "articles" and "reviews". All studies published from 1st January 1995–31 st December 2024, and the literature search and extraction of data were completed on January 6th, 2025.

### Literature screening and data extraction

**Fig. 1:**
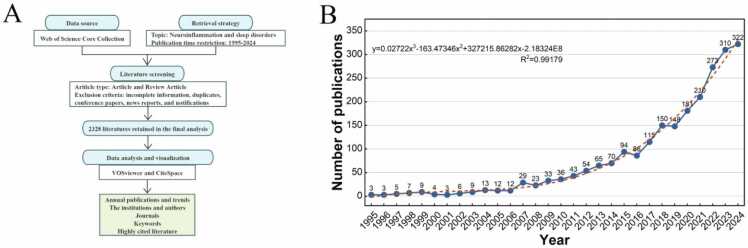
The flow chart for literature screening and annual publication trends. The flow chart for literature screening. (B) Annual publication trends.

To ensure the reliability of the results, literature screening and data extraction were conducted independently by two researchers. Selected literature was downloaded in "plain text" format into Note Express software, collecting basic information such as publication year, country/region, institution, author, journal, and keywords for further analysis. Exclusion criteria included incomplete author and institution information, unclear publication years, incomplete keywords, and duplicate publications. Additionally, duplicates, conference papers, news reports, and notifications were manually removed. The overall process of literature screening and data extraction is illustrated in Fig. 1A.

### Data analysis and visualization

The literature filtered by the database is exported in Refworks format and named 'download-xx.txt'. Use NoteExpress3.6.0 software to conduct annual science frequency statistics on the included literature, and use Origin2024 to draw a line graph of publication volume. Then import the file in 'download. txt' format into VOSviewer 1.6.20 and CiteSpace 6.1.R6 software. This study utilized VOSviewer software to analyze and visualize country or regional distribution, author collaboration and distribution, institution collaboration and distribution, the journals and co-cited journals, references citation and co-citation, and keyword distribution and collaboration. Simultaneously, this study used CiteSpace to analyze and visualize the co-citation of references, the dual map overlay of journals, and keyword bursts. In these visualized graphs, the node diameter size and the name font size represent the frequency of occurrence, with higher frequency resulting in larger nodes and font sizes. The connection and the thickness between the nodes represent the co-occurrence relationship and their association strength. Node colors represent the different years. Centrality is one of the important indicators for measuring the importance of a node in a graph, with higher centrality indicating that the node is more important in the field.

## Results

### Annual publications and trends

To a certain extent, the number of annual publications can indicate the research level and overall trends of the research field. Fig. 1B illustrates the annual output and trends of the literature published over the past three decades. In terms of annual publications, the development of the research field on sleep disorders and neuroinflammation revealed three different stages. Stage one (1995–2006) marked the Enlightenment era, during which the number of relevant literature was relatively limited. Stage two (2007–2016) was a slow development period in this field, with a gradual increase in the number of publications, indicating that this field received attention from researchers during this period. Stage three (2016–2024) was in a period of rapid expansion, with the most active research heat and a clear trend of increasing annual publication output. This indicates that researchers have gained further understanding of the field and research has gradually become more comprehensive.

### Analysis of countries and regions

**Fig. 2:**
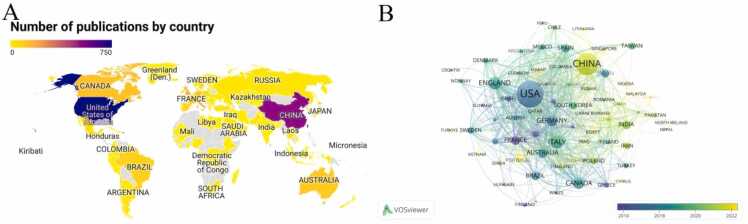
Geographical distribution and network of countries/regions. Geographical map shows the global distribution of research. (B) Visualization of countries/regions cooperation network. (This network includes 68 nodes (countries/regions) with 655 edges (co-occurrence links). The network was constructed using a threshold that retains all countries with at least one co-occurrence relationship, ensuring comprehensive coverage of global research collaboration patterns.).

VOSviewer was employed to visualize the publication and cooperation status of countries or regions around the world. As shown in Table 1 and Fig. 2A, a total of 68 countries/regions worldwide have contributed to research on sleep disorders and neuroinflammation. The United States has the highest number of publications (750), followed by China (523), Italy (175), the United Kingdom (163) and Canada (128). It was worth noting that the United States was a research center with significant influence in this field and maintained close cooperative relationships with numerous countries/regions. In the overlay visualization, different colors represented different annual distributions based on the concentrated year of publication by countries/regions, with a preference for yellow indicating the most recent publication. From Fig. 2B, it could be seen that the main contributions of China, Saudi Arabia, Portugal and Egypt primarily focused on recent publications, have become active in recent years, while France, Switzerland and Finland were more prominent in the early years of the field.

**Table 1:** Top 20 countries/regions based on publications.

**Rank**	**Country/Region**	**Documents**	**Citation**	**Centrality**	**Average citations**
1	USA	750	42855	0.09	57.14
2	CHINA	523	8550	0.04	16.35
3	ITALY	175	8407	0.07	48.04
4	ENGLAND	163	7864	0.18	48.25
5	CANADA	128	8293	0.09	64.79
6	GERMANY	120	7582	0.3	63.18
7	AUSTRALIA	100	4982	0	49.82
8	FRANCE	87	6655	0.32	76.49
9	INDIA	81	1972	0	24.35
10	BRAZIL	79	2446	0	30.96
11	SPAIN	71	2816	0.01	39.66
12	JAPAN	69	1436	0.23	20.81
13	SOUTH KOREA	56	1343	0	23.98
14	TAIWAN	55	961	0	17.47
15	NETHERLANDS	47	3397	0.49	72.28
16	SWEDEN	46	2185	0.12	47.50
17	IRAN	45	935	0	20.78
18	POLAND	45	1467	0.06	32.60
19	SWITZERLAND	39	2263	0.09	58.03
20	DENMARK	35	2457	0.1	70.20

### Analysis of the institutions and authors

**Fig. 3:**
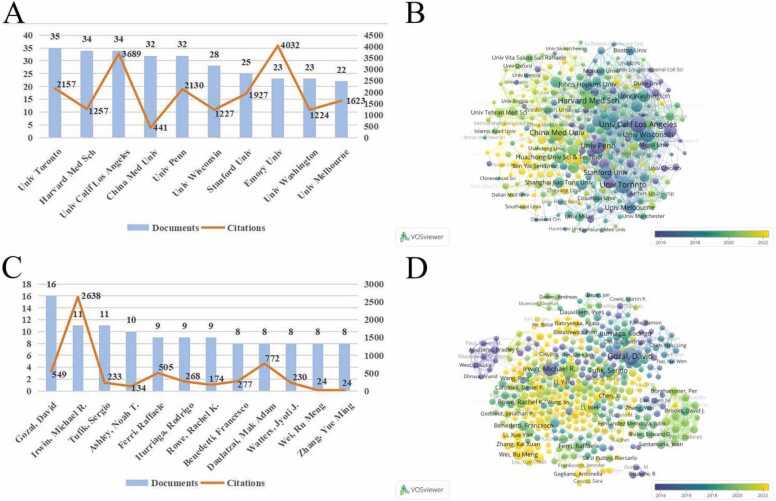
The distribution of institutions and authors. (A) The top 10 institutions with the most publications. (B) Collaborative networks of institutions based on publication chronological order. (The network comprises 283 nodes (institutions) connected by 1196 edges. Nodes represent institutions with ≥ 5 publications in the field, and edges reflect co-authorship collaborations with a similarity threshold of 0.1, balancing granularity and interpretability.) (C) The top 12 authors with the most publications. (D) Collaborative networks of authors based on publication chronological order. (This network includes 374 nodes (authors) and 705 edges. Authors with ≥ 3 publications were included as nodes, and edges were retained for co-authorship relationships with a strength ≥ 2, focusing on core contributors and their collaborative ties.).

Over the past thirty years, many institutions and their scholars have made tremendous efforts to promote the development of this field. Among them, there were 283 institutions with more than 5 publications, and the top 10 institutions with the highest publication output were listed in Fig. 3A. Among these institutions, 7 out of 10 were from the United States, reflecting the excellent research capabilities of the United States in this field in terms of both publication output and citation frequency. The top five institutions in terms of publication output were *Univ Toronto* (n = 35), *Harvard Med Sch* (n = 34), *Univ Calif Los Angeles* (n = 34), *China Med Univ* (n = 32), and *Univ Penn* (n = 32). In order to understand the collaborative relationships between institutions and the relationship between the number of publications and the timeline, we used VOSviewer for visual analysis. The size of network nodes and fonts represents the number of publications, with larger ones indicating a higher total number of publications. The connections between nodes represent the level of collaboration. In addition, the different colors of nodes can reflect the average publication year of various institutions in the field. AS shown in Fig. 3B, we could see that research institutions with a high number of publications have shown close collaborative relationships. Research institutions represented by *Univ Penn* and *Univ Calif Los Angeles* were early pioneers in this field. Instead, researchers from *China Med Univ* were active in recent years. Instead, research institutions represented by *China Med Univ* have been very active in this field in recent years. Next, we analyzed the major authors. The top 12 most prolific authors were listed in Fig. 3C (due to the same number of articles published by authors ranked 8–12, all of them were included, resulting in 12 authors being listed). Among them, Gozal, David (n = 16), Irwin, Michael R. (n = 11), Tufik, Sergio (n = 11), and Ashley, Noah T. (n = 10) had published over 10 articles, indicating that they were the most influential authors in the field. From the collaborative network diagram of the authors, we could see that most researchers who had published more papers had illustrated good cooperative relationships, and the edge nodes in the diagram represented independent scholars who had achieved certain results in the field. Although they lacked close collaboration with active teams, they still had some influence in this field. At the same time, we could see that the research teams led by Gozal, David and Irwin, Michael R. were pioneers in this field, signifying their significant contributions to the development and progress of sleep disorders and neuroinflammation. In contrast, Chinese researchers such as Wei, Ru Meng, Zhang, Yue Ming and Chen, Xi, etc. were highlighted in yellow, indicating a significant increase in research participation or publication output in this field in recent years (Fig. 3D).

### Analysis of journals

**Fig. 4:**
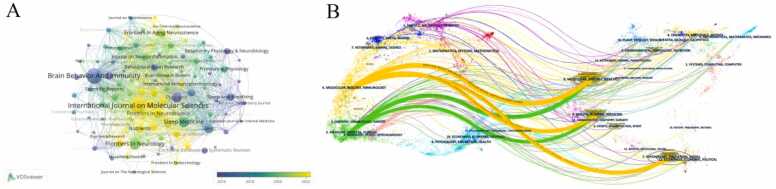
The distribution of Journal. (A) Collaborative networks of journals based on publication chronological order. (The network contains 108 nodes (journals) and 986 edges. Journals with ≥ 10 publications in the dataset were included, with edges representing co-citation relationships (minimum co-citation count = 5), highlighting key academic platforms in the field.) (B) A double-map overlay of journals.

The visualization analysis of journal distribution could provide valuable insights for researchers in this field to consult literature and target journals for publishing papers. The results showed that 108 journals had published at least 5 articles, and Table 2 listed the top 10 journals ranked by article output. The *International Journal on Molecular Sciences* and *Brain Behavior And Immunity journals* in the United States had similar publication output. The former ranked first with a total output of 55 articles, but the latter was more influential in terms of article citation count. The research interests of these journals mainly focus on medicine, biology, and neurology. Five publishers are from Switzerland, four from the US, and another publisher from the Netherlands. We also used VOSviewer to visually analyze the journals, and Fig. 4A shows the publication output and average publication year of different journals. Among them, *Brain Behavior And Immunity* had published earlier and had a significant impact in this field. Journals represented by the *International Journal on Molecular Sciences* had published a large number of articles related to this field in recent years, which deserves the attention of researchers in this field. The colored paths on both sides of the overlay map depict the citation relationships between different fields, and the dual-map overlay display of journal publications helps researchers understand the interrelationships and hot topics between different disciplines in the field. In the dual-map overlay published in journals in this field (Fig. 4B), there was an orange main citation path and a green main citation path. Molecular/Biological/Immunology journals were represented by orange paths, while Medicine/Medical/Clinical journals were represented by green paths. The orange and green pathways were both cited in the fields of Molecular/Biology/Genetics and Health/Routing/Medicine. In addition, the orange path was also related to the field of Psychology/Education/Social.

**Table 2:** Top 10 journals in terms of the number of publications.

**Rank**	**Journals**	**Articles**	**Citations**	**Region**	**JCR-C**	**IF(2023)**
1	International Journal on Molecular Sciences	55	1687	US	Q2	4.9
2	Brain Behavior And Immunity	53	3070	US	Q2	8.8
3	Frontiers In Neurology	33	884	Switzerland	Q3	2.7
4	Sleep Medicine	31	622	Netherlands	Q2	3.8
5	Plos one	28	1336	US	Q3	2.9
6	Frontiers In Neuroscience	26	308	Switzerland	Q3	3.2
7	Sleep	24	889	US	Q2	5.3
8	Frontiers In Aging Neuroscience	23	731	Switzerland	Q2	4.1
9	Brain Sciences	22	280	Switzerland	Q3	2.7
10	Nutrients	22	331	Switzerland	Q2	4.8

### Analysis of keywords

**Fig. 5:**
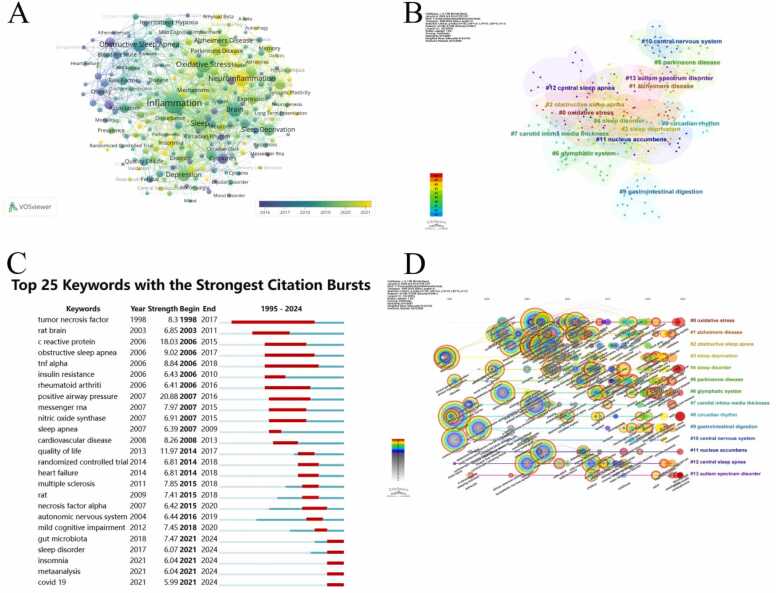
The analysis of keywords. (A) Collaborative networks of keywords based on publication chronological order. (This network consists of 232 nodes (keywords) and 12,735 edges. Keywords with ≥ 15 occurrences were included, and edges reflect co-occurrence frequency with a similarity threshold of 0.05, capturing the dense interconnectedness of research topics.) (B) The visualization map of the keywords clustering analyses. (The network parameters are: N = 349 nodes, E = 550 edges (network density=0.0091). Pruning was performed using the Pathfinder algorithm. Modularity Q= 0.8007 (indicating strong clustering structure), Weighted Mean Silhouette S= 0.9139 (indicating high cluster homogeneity), and Harmonic Mean(Q,S)= 0.8536, confirming the robustness of the clustering results.) (C) Top 25 keywords with the strongest citation bursts. (D) The timeline view of the cluster analysis for keywords from 1995 to 2024.

Keywords are an important component of an article, and co-occurrence and clustering analysis can help to systematically and quickly understand the research hotspots and directions in this field (Li and Wu, 2024). We utilize VOSviewer to analyze keywords that appeared ≥ 20 times in the entire literature, and used different colors to represent research hotspots in different periods. Purple represented early popular research topics, while yellow represented recent research hotspots (Fig. 5A). In this analysis, a total of 232 keywords were identified and divided into 14 clusters, including: #0 oxidative stress、#1 alzheimers disease、#2 obstructive sleep apnea、#3 sleep deprivation、#4 sleep disorder、#5 parkinsons disease、#6 glymphatic system、#7 carotid intima media thickness、#8 circadian rhythm、#9 gastrointestinal digestion、#10 central nervous system、#11 nucleus accumbens、#12 central sleep apnea、#13 autism spectrum disorder (Fig. 5B).

At the same time, keyword burst analysis was performed based on CiteSpace, the top 25 burst keywords were shown in Fig. 5C. Year represents the year of its first appearance, Strength represents its emergence rate, the red band represents a sudden increase in the use of this keyword during this period, and blue and light blue represent relative unpopularity. In the past three decades, the strongest citation burst keyword was positive airway pressure (20.88), followed by c reactive protein (18.03) and quality of life (11.97). Meanwhile, some keywords burst with long time, such as tumor necrosis factor, tnf alpha and obstructive sleep apnea (OSA), with a time span of 19, 12 and 11 years, respectively. The results of keyword emergence based on the timeline showed that the research on gut microbiota, sleep disorder, insomnia, metaanalysis and covid 19 have been research hotspots in recent years, indicating potential research hotspots in this field in the coming years.

The time line visualization of keywords could display the development and evolution of research hotspots under the cluster based on the time span, and revealed the emergence of new hotspots. It helps scholars to enhance their perception of the research field and improve their understanding of the forefront of research (Huang et al., 2024). By observing Fig. 5D, it could be observed that oxidative stress, Alzheimer's disease (AD), and sleep disorder appeared early and lasted for a long time, indicating that they have always been hot topics in this research field. Although OSA and CNS appeared early, their attention has gradually decreased in recent years. Although sleep deprivation (SD), Parkinson's disease (PD), glymphatic system and gastrointestinal digestion went unnoticed for nearly a decade, their research heated has rebounded in recent years.

### Analysis of highly cited literature

**Fig. 6:**
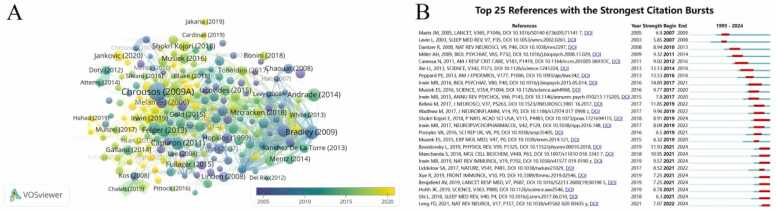
The analysis of highly cited literature. (A) Collaborative networks of highly cited references based on publication chronological order. (This network encompasses 194 nodes (individual literatures) and 2231 edges (reflecting literature coupling relationships). For inclusion, a targeted threshold was applied: only literatures with a citation count of ≥ 100 were incorporated. Focusing on highly - cited works (≥100 citations) highlights the core “knowledge backbone” of the field.) (B) Top 25 references with the strongest citation bursts.

The citation frequency of literature can reflect its academic influence and extensive research interests. Highly cited literature usually receives significant scholarly attention in the field and reveals research hotspots in the field, which has guiding significance for subsequent research (Zhang et al., 2024). We conducted a statistical analysis of 2328 articles and found that 199 of them were cited more than 100 times. Fig. 6A presented the network and density visualization of the co-cited references. Table 3 summarizes the top 20 most cited articles in the fields of sleep disorders and neuroinflammation. As shown in the table, the citation frequency of these articles ranged from 445 to 2046 times. And most of them were published in Q1 journals (80 %), indicating that they are highly influential or pioneering in this field. The most cited article, with 2046 citations, is a review published by Chrousos GP et al. in 2009 in *Nat Rev Endocrinol*(Chrousos, 2009). The article mentions that when the homeostasis of an organism is threatened or perceived as threatened, it can trigger a stress response. Malfunctions in the stress system may impair growth, development, behavior, and metabolism, leading to various acute and chronic diseases. The article ranked second in citation count was a research article published in the *Lancet* in 2009 (Bradley and Floras, 2009). This paper expounds the relationship between OSA and cardiovascular diseases, and reviews the role of diagnosis and treatment of OSA in reducing the incidence rate and mortality of cardiovascular diseases. Fig. 6B illustrated the top 25 references with the strongest citation bursts. The result indicate that astrocytes, microglia, sleep-immune crosstalk and sleep-wake cycle were hotspots in current research.

**Table 3:** The top 20 highest-cited articles.

**Rank**	**Title**	**First author** **(year)**	**Journal** **(JCR-C, IF)**	**Total citations**
1	Stress and disorders of the stress system (Chrousos, 2009)	Chrousos GP (2009)	Nat Rev Endocrinol (Q1, 31)	2046
2	Obstructive sleep apnoea and its cardiovascular consequences (Bradley and Floras, 2009)	Bradley TD (2009)	Lancet (Q1, 98.4)	1008
3	The clinical profile and pathophysiology of atrial fibrillation: relationships among clinical features, epidemiology, and mechanisms (Andrade et al., 2014)	Andrade J (2014)	Circ Res (Q1, 16.5)	870
4	Immune system to brain signaling: neuropsychopharmacological implications (Capuron and Miller, 2011)	Capuron L (2011)	Pharmacol Ther (Q1, 12)	828
5	Inflammatory cytokines in depression: neurobiological mechanisms and therapeutic implications (Felger and Lotrich, 2013)	Felger JC (2013)	Neuroscience (Q3, 2.9)	775
6	Sleep apnoea as an independent risk factor for cardiovascular disease: current evidence, basic mechanisms and research priorities (McNicholas and Bonsigore, 2007)	McNicholas WT (2007)	Eur Respir J (Q1, 16.6)	698
7	Reciprocal regulation of the neural and innate immune systems (Irwin and Cole, 2011)	Irwin MR (2011)	Nat Rev Immunol (Q1, 67.7)	631
8	Burnout and risk of cardiovascular disease: evidence, possible causal paths, and promising research directions (Melamed et al., 2006)	Melamed S (2006)	Psychol Bull (Q1, 17.3)	600
9	Parkinson's disease: etiopathogenesis and treatment (Jankovic and Tan, 2020)	Jankovic J (2020)	J Neurol Neurosurg Psychiatry (Q1, 8.7)	548
10	β-Amyloid accumulation in the human brain after one night of sleep deprivation (Shokri-Kojori et al., 2018)	Shokri-Kojori E (2018)	Proc Natl Acad Sci (Q1, 9.4)	513
11	What we know about primary dysmenorrhea today: a critical review (Iacovides et al., 2015)	Iacovides S (2015)	Hum Reprod Update (Q1, 14.8)	504
12	Depressive disorders and immunity: 20 years of progress and discovery (Irwin and Miller, 2007)	Irwin MR (2007)	Brain Behav Immun (Q2, 8.8)	502
13	Mechanisms linking circadian clocks, sleep, and neurodegeneration (Musiek and Holtzman, 2016)	Musiek ES (2016)	Science (Q1, 44.7)	500
14	Sleep and athletic performance: the effects of sleep loss on exercise performance, and physiological and cognitive responses to exercise (Fullagar et al., 2015)	Fullagar HH (2015)	Sports Med (Q1, 9.3)	486
15	Physiology of the prion protein (Linden et al., 2008)	Linden R (2008)	Physiol Rev (Q1, 29.9)	484
16	Neuropsychological sequelae and impaired health status in survivors of severe acute respiratory distress syndrome (Hopkins et al., 1999)	Hopkins RO (1999)	Am J Respir Crit Care Med (Q1, 19.3)	478
17	Neuropathic pain: mechanisms and their clinical implications (Cohen and Mao, 2014)	Cohen SP (2014)	BMJ (Q1, 93.6)	467
18	Neuroinflammation as a Common Feature of Neurodegenerative Disorders (Guzman-Martinez et al., 2019)	Guzman-Martinez L (2019)	Front Pharmacol (Q2, 4.4)	465
19	Pathophysiology of the metabolic syndrome (McCracken et al., 2018)	McCracken E (2018)	Clin Dermatol (Q3, 2.3)	458
20	Perivascular spaces in the brain: anatomy, physiology and pathology (Wardlaw et al., 2020)	Wardlaw JM (2020)	Nat Rev Neurol (Q1, 28.2)	445

## Discussion

This study reviewed and analyzed relevant literature on neuroinflammation and sleep disorders from the WoSCC database over the past 30 years using VOSviewer and CiteSpace software, but there are several limitations to this research. First, it only includes literature from the WoSCC database, which may overlook high-quality studies in other databases such as Scopus and PubMed. Second, we only included English-language literature, which may neglect valuable non-English publications. Additionally, earlier literature tends to have higher citation rates, which could result in newly published high-quality studies being overlooked due to their lower citation counts. While these limitations are difficult to avoid, bibliometric analysis can systematically analyze and visualize the research landscape and hotspots in a specific field, and it also helps identify new research trends, providing a valuable tool for newcomers in this area.

The number of publications and citation counts are important indicators for assessing the academic quality and influence of countries, institutions, or individuals. Through retrieval and analysis, it is evident that the overall number of published papers has shown an upward trend over the past 30 years, particularly in the last decade, where there has been a significant increase in publications in this field. It is expected that interest in research in this area will remain strong, and the number of publications will continue to grow, leading to more in-depth studies. The United States and China have the highest number of publications, indicating their significant contributions to this research field. In contrast, developed countries such as Germany, France, and Italy exhibit higher centrality and average citation rates, suggesting that these countries also play a leading role in the academic development of this field. Therefore, these results indicate that publication volume does not fully represent academic influence; countries should place greater emphasis on the creativity and technological innovation of research, strengthen collaboration, and enhance academic impact. Among the top 10 institutions, 7 are from the United States, while the remaining 3 are from Canada, China, and Australia. The substantial output from these research institutions reflects adequate financial support from these countries and the ongoing attention of elite scholars. Notably, the *University of California, Los Angeles* (34 publications, 3689 citations) and *Emory University* (23 publications, 4032 citations) in the U.S. not only have a high volume of publications but also demonstrate significant influence.

Gozal, David, Irwin, Michael R, and Tufik, Sergio have made significant contributions to the progress of research on neuroinflammation and sleep disorders. Research conducted by the world-renowned pediatric sleep expert Dr. David Gozal and others indicates that chronic fragmented sleep can lead to cognitive decline, increased expression of inflammatory markers such as NF-κB, TNF-α, and IL-1β, as well as heightened activation of microglia. Furthermore, the researchers have demonstrated that the cognitive impairment caused by fragmented sleep is closely related to changes in the integrity of the blood-brain barrier (Puech et al., 2023). The University of California's Institute for Neuroscience and Human Behavior, represented by Michael R. Irwin, has been dedicated to researching the relationship between sleep disorders and the activation of inflammatory cells for many years. Studies have shown that different stages of sleep have varying regulatory effects on the activity of inflammatory cytokines. Sleep disorders activate the NF-κB, AP-1, and STAT signaling pathways, increasing the production of IL-6 and TNF by monocytes stimulated by TLR4, which leads to sustained activation of inflammatory responses and contributes to the development of diseases such as depression, dementia, cardiovascular diseases, and certain cancers (Irwin, 2019, Piber et al., 2022, Manigault et al., 2021). In terms of journals, the main publications in the field included *International Journal on Molecular Sciences* (IF = 4.9, Q2) and *Brain Behavior And Immunity* (IF = 8.8, Q2) based on the number of publications, while *Brain Behavior And Immunity* was the most cited journal. The research topics in these leading journals primarily include the pharmacological alleviation of inflammation caused by SD (Engert et al., 2024, Ballesio, 2024), the mechanisms by which gut microbiota dysbiosis affects sleep disorders (Sun et al., 2023, Zhao et al., 2023), and the impact of sleep disorders on the activation of glial cells and inflammasomes (Ni et al., 2024). In contrast, journals such as *Frontiers in Neurology* and *Sleep Medicine* focus more on the diagnosis, clinical research, and biomarkers of sleep disorders (Andersen et al., 2024, Liu et al., 2023, Zeng et al., 2025, Tao et al., 2024). Notably, according to the JCR 2023 standards, the majority of the journals with the highest publication volume are classified as Q2 or Q3. This suggests that researchers in this field still need to enhance the innovation and impact of their studies and publish more high-quality, high-impact papers.

Keywords and their clustering represent a distillation of the core content of the text. The analysis results indicate that oxidative stress, inflammation, cytokines, and microglia have been focal points of research over the past 30 years. Sleep disorders are a public health epidemic that affects individuals across all age groups, particularly older adults who are more susceptible. With the increasing aging population, the prevalence of sleep disorders is rising year by year. Sleep disorders not only disrupt multiple systems in the body, including the immune, endocrine, and nervous systems, but also contribute to or exacerbate the occurrence and progression of various diseases. Similarly, sleep disorders are common and exacerbating manifestations of several neuroimmune diseases, such as AD, PD, and autism (Xiang et al., 2024, Nogueira et al., 2023, Zahed et al., 2021). While the importance of the interplay between sleep disorders and neuropsychiatric diseases is indisputable, the underlying pathophysiological mechanisms remain controversial due to the involvement of interactions among multiple organs and pathways. Increasing evidence suggests that oxidative stress and inflammation appear to be common factors in the pathophysiology of sleep disorders. They can influence the expression of circadian rhythm regulatory factors by activating microglia to secrete pro-inflammatory cytokines. Simultaneously, SD disrupts the body’s immune balance and is associated with dysregulation of the inflammatory process, further exacerbating oxidative stress and neuroinflammation in the physiological cycles of severe cases of neuroimmune diseases. Microglia are the resident immune cells in the brain and have garnered widespread attention due to their crucial roles in brain development, neuronal protection, and synaptic plasticity. In recent years, the activation of microglia has been considered a major source of neuroinflammation and a key factor in regulating sleep disorders (Gu et al., 2023, Parhizkar et al., 2023, Liu et al., 2024). The activation of microglia caused by sleep disorders not only leads to the onset of neuroinflammation but also enhances their phagocytic activity, damages synaptic pruning, and increases the brain's susceptibility to other forms of injury. Meanwhile, it can also affect the autophagic function of microglial mitochondria, thereby inducing an imbalance in genes associated with neuronal apoptosis (Xiong et al., 2024). The pathways and mechanisms by which sleep disorders activate microglia are complex and diverse, and the regulatory roles of various signaling pathways in sleep have received extensive attention and comprehensive study. In mice subjected to chronic sleep deprivation (CSD), the extracellular concentration of ATP in the prefrontal cortex significantly increases, thereby activating the P2X7 receptors on the surface of glial cells, which stimulates the release of sleep-regulating factors TNF-α and IL-1β, enhancing neuroinflammation (Xia et al., 2020, Li et al., 2023). Research by Fan et al. found that SD can induce the activation of hippocampal microglia and phosphorylation of p38 MAPK, with significant increases in NLRP3 inflammasome levels and inflammatory factors IL-1β and IL-18 in the hippocampus. Notably, recovery sleep effectively inhibits the activation of the p38 MAPK signaling pathway (Fan et al., 2021). Additionally, microglia can lead to the release of inflammatory cytokines and chemokines through the activation of pathways such as TLR4/NF-κB, JAK/STAT, and α7-nAChR (Lim et al., 2025, Hsiao et al., 2024, Atta et al., 2023).

Cytokines are polypeptide proteins produced by immune cells that can regulate various cell functions through multiple mechanisms (Oppenheim, 2018). As molecular messengers of both innate and adaptive immunity, they enable communication among immune system cells via paracrine and autocrine signaling. Over the past thirty years, exploring the interactions between pro-inflammatory cytokines and sleep disorders has been a focal point of research in this field (Irwin, 2019, Opp, 2005). It is well known that inflammatory cytokines in the CNS, such as high-sensitivity C-reactive protein, Interleukin (IL)-1, Interleukin (IL)-6, and Tumor Necrosis Factor (TNF), can regulate sleep (Akkaoui et al., 2023, Chuang et al., 2024). At the same time, SD can lead to significant alterations in the immune response system. In contrast, other inflammatory cytokines have received less attention. The body's immune-inflammatory response is regulated by a complex immune network and various inflammatory factors, and the interplay and mutual inhibition between pro-inflammatory cytokines and anti-inflammatory cytokines are closely related to the occurrence of sleep disorders (Sang et al., 2023, Besedovsky et al., 2019a). The application of anti-inflammatory cytokines as a potential treatment for sleep disorders may become one of the avenues for future research exploration, offering valuable insights for upcoming clinical studies.

Highly cited literature often reflects the hotspots and advancements in a research field. The two most frequently cited articles were published in 2009, which we have already summarized earlier. Next, we will focus on highly cited articles from recent years. A review by Jankovic J and Tan EK published in 2020 in *J Neurol Neurosurg* emphasizes that sleep disorders are prodromal symptoms of PD, developing alongside cognitive impairment and autonomic dysfunction as the disease progresses. The article points out that key pathogenic mechanisms of PD are closely related to neuroinflammation and oxidative stress (Jankovic and Tan, 2020). A groundbreaking clinical study by Shokri-Kojori E et al. published in 2018 provided evidence that acute sleep deprivation (ASD) may affect the clearance of β-amyloid (Aβ) in the human brain, highlighting the importance of good sleep in maintaining normal brain function and preventing AD (Shokri-Kojori et al., 2018). The citation burst intensity was strongest for a review by Irwin MR published in 2016 in *Biol Psychiatry* (14.89, 2017–2021), which analyzed 72 studies and found that sleep disorders and prolonged sleep duration are associated with increased levels of systemic inflammatory markers (Irwin et al., 2016). In an article published by Besedovsky L in 2019, the bidirectional relationship between sleep and immunity was emphasized, and the summary of previous studies suggested that the impact of sleep on the immune system is likely mediated by influencing several inflammatory mediators, such as cytokines, to promote inflammatory homeostasis (Besedovsky et al., 2019b). In recent years, a study by Manchanda S et al. published in 2018 (10.95, 2021–2024) has shown a strong citation burst in basic research. This study found that CSD leads to increased levels of inflammatory cytokines (TNFα, IL-1β) in the hippocampus and piriform cortex of rats, activation of the transcription factors NFκB and AP1, along with enhanced expression of GFAP and Iba1 in both brain regions (Manchanda et al., 2018).

Through the study of literature on neuroinflammation and sleep disorders, we found that recent research in this field has primarily focused on the deep exploration of the interaction mechanisms between the two, the comorbidity of neural circuits related to sleep disorders and psychiatric diseases, the search for specific biomarkers, and intervention strategies aimed at alleviating sleep disorders by improving neuroinflammation (Dauvilliers, 2025, Giri et al., 2024, Tamir et al., 2023, Heneka et al., 2024). While this area has garnered widespread attention from researchers and relevant studies are being actively conducted, the complexity of interactions among multiple systems and organs in the human body, along with the unclear sharing of pathways among various neuropsychiatric diseases, indicates that there are still many gaps and challenges in the research. In our literature review, we found that most studies are primarily conducted using animal models. Undoubtedly, these studies are crucial for exploring the role of neuroinflammation in the onset and progression of sleep disorders. However, significant differences exist between human brain tissues and those of rodents or primates in terms of genetics, cell types, and epigenetic information. The existing models struggle to simulate the chronic processes of human sleep-inflammation interactions, which greatly restricts the translation of experimental results from animal studies to clinical applications (Lancaster and Knoblich, 2014, Zhao and Bhattacharyya, 2018, Dawson et al., 2018). In recent years, the emergence of human brain organoids has addressed the limitations of traditional models and has facilitated research on human brain development and psychiatric disorders (Schutgens and Clevers, 2020). Brain organoids refer to three-dimensional cell complexes that resemble human target organs, formed in vitro by inducing the differentiation of embryonic stem cells, pluripotent stem cells, or adult stem cells. They exhibit cellular diversity and electrophysiological activity, allowing them to simulate the growth and development processes of the early human brain at the molecular, cellular, and structural levels, and they possess physiological functions similar to those of the human brain (Chiaradia et al., 2023, Wang et al., 2023, Sandoval et al., 2024). As a promising tool for studying human brain development and function, human brain organoid models are now widely used in various fields, including neurodegenerative diseases, neurodevelopmental disorders, and drug addiction (Chang et al., 2020, Dionne et al., 2025, Adams et al., 2023). With the advancement of science and technology, there is potential for integration with emerging technologies such as gene editing and organ-on-a-chip systems, which may help overcome the limitations of traditional disease research models and provide broader prospects for translational medicine in this field.

## Conclusion

This article presents a bibliometric analysis of research on neuroinflammation and sleep disorders over the past 30 years, offering visual insights from various aspects such as countries, institutions, authors, journals, and keywords. Overall, this research field is in a developmental stage and shows potential for continuous expansion. The United States and China are the largest contributors in this area, with major research institutions in the U.S. consistently leading global research efforts. With the development of technologies such as single-cell sequencing, gene editing, and organoid transplantation, along with the establishment of multidisciplinary collaborative networks integrating sleep medicine, immunology, and computational biology, we anticipate that exploring deeper molecular mechanisms and the application of organoids to elucidate the dynamic effects of neuroinflammation in the sleep-wake cycle may become promising research avenues in this field. In conclusion, I believe that the results of this study can provide valuable references for researchers in this area.

## CRediT authorship contribution statement

**Zhao-qiong Zhu:** Writing – review & editing. **Nan Zhao:** Writing – original draft, Conceptualization. **Qihai Gong:** Writing – review & editing. **Rui Jiang:** Writing – original draft.

## Informed consent statement

Not applicable.

## Institutional review board statement

Not applicable.

## Funding

This study was supported by the Zunyi Science and Technology Cooperation Fund (No. HZ2022–391), the Guizhou Provincial Health Commission Science and Technology Fund Project (No. gzwkj2022–381), and the Guizhou Provincial Administration of Traditional Chinese Medicine (No. QZYY-2021–111).

## Declaration of Competing Interest

The authors declare that they have no known competing financial interests or personal relationships that could have appeared to influence the work reported in this paper.

## Data Availability

The original contributions presented in the study are included in the article/Supplementary material, further inquiries can be directed to the corresponding author.
